# Corynebacterium Bovis: A Rare Case of Persistent Bacterial Keratitis and Corneal Perforation

**DOI:** 10.7759/cureus.16913

**Published:** 2021-08-05

**Authors:** Mohammed Elsheikh, Ahmed Elsayed, Nicholas Bennett, Martin Connor

**Affiliations:** 1 Ophthalmology, NHS Dumfries & Galloway, Dumfries, GBR; 2 Microbiology, NHS Dumfries & Galloway, Dumfries, GBR

**Keywords:** corynebacterium bovis, cornea, microbial keratitis, corneal perforation, corneal infection

## Abstract

We report a rare case of severe, non-contact lens-related *Corynebacterium bovis* corneal infection on a background of viral keratitis, resulting in corneal abscess formation with subsequent corneal perforation. An 89-year-old Caucasian lady presented with a significant epithelial defect and a dense stromal infiltrates on a background of herpes zoster keratitis, ultimately resulting in corneal perforation. Enrichment culture obtained from corneal scraping isolated the unusual organism *Corynebacterium bovis*. This was treated with a combination of culture-directed, targeted course of antibiotics and surgical interventions. To the best of our knowledge, this is the first reported case of profuse bacterial keratitis secondary to *Corynebacterium bovis* infiltration, on a background of viral keratitis, resulting in corneal abscess formation and subsequent perforation. This report highlights this rare bacterium’s characteristics including its pathogenicity in causing severe corneal disease, particularly in immunosuppressed environments such as in this case, apparent antibiotic sensitivities & resistance, and potential transmission route.

## Introduction

Corynebacterium species are frequently isolated on the conjunctiva of healthy adults and are thus increasingly regarded as non-pathogenic bacteria [1,2]. Recently, however, there have been increasing reports detailing the pathogenicity of Corynebacterium species isolated on ocular surfaces, with immunosuppression representing a risk factor [3-6].

*Corynebacterium bovis* is an established bovine commensal bacterium, commonly resulting in bovine mastitis [7]. Infections in humans caused by *Corynebacterium bovis* remain extremely rare, with only nine cases reporting non-ocular infections [8-11] and four cases reporting ocular involvement [12]. These four cases reported involvement of predominantly the eyelid, conjunctiva and in one case, a cheek wound infiltration with subsequent facial soft tissue extension. Herein, we present the first reported case of severe bacterial keratitis secondary to *Corynebacterium bovis* infiltration, on a background of viral keratitis, resulting in corneal abscess formation and subsequent perforation.

## Case presentation

An 89-year-old female with no relevant past medical history was referred to our centre on 9th January 2020 with reduced vision in her right eye and associated significant pain for 3 days. The best-corrected visual acuity (BCVA) was noted to be 0.36 logMAR in the right eye and 0.24 logMAR in the left eye. Slit-lamp biomicroscopic examination revealed right-sided periorbital erythema, conjunctival injection, a deep and quiet anterior chamber, and a clear cornea. She was also noted to have a vesicular lesion overlying her right eyebrow. Intraocular pressure was noted to be within normal limits in both eyes, and the right eye posterior segment examination was also normal. The left eye anterior and posterior segment examination was normal. In view of these examination findings, a working diagnosis of herpes zoster ophthalmicus was made, and the patient was prescribed high-dose acyclovir tablets (800 mg five times per day) and topical ganciclovir 0.15% gel three times per day.

By 4th March 2020, the BCVA in the affected eye had further deteriorated to 0.74 logMAR and anterior segment examination revealed persisting conjunctival injection and superficial punctate keratopathy, with the development of an inferior corneal epithelial defect measuring approximately 2 mm x 3 mm, confirmed with fluorescein staining. The patient was prescribed topical levofloxacin hourly. On clinic review two weeks later, the inferior epithelial defect had slightly increased in size, and 2+ inflammatory cells were observed in the anterior chamber. Due to this size progression, her treatment was switched from topical levofloxacin to chloramphenicol ointment three times daily, to provide further anti-microbial coverage. In view of the anterior chamber reaction, we also recommenced topical prednisolone sodium phosphate 0.5% three times daily, with the addition of a topical lubricating gel to be used up to 6 times daily.

By 28th April 2020, the anterior chamber reaction had largely resolved. However, the inferior corneal epithelial defect remained unchanged, prompting cessation of steroid medication, the commencement of preservative-free eye drop formulations, and continuing the generous use of topical lubricating agents, to promote healing. A bandage contact lens and a protective plastic shield were also utilised to encourage healing of the persistent inferior epithelial defect. However, despite these attempts, there was negligible healing of the epithelial defect. On the next clinic review, the epithelial defect now measured approximately 3 mm x 0.7 mm, and inferior stromal keratolysis with thinning was noted. Corneal sensation was also dramatically reduced in this eye.

On August 3rd, the BCVA had deteriorated to hand movements. On examination, a superior white dense corneal abscess was visualised. At this point, we sent a corneal scraping to the microbiology department for culture and arranged for urgent hospital admission. Corneal scrapings were inoculated on chocolate agar, Sabouraud dextrose agar, blood agar, starch ampicillin agar, and starch ampicillin agar with neomycin. Hourly topical ciprofloxacin 5% and hourly topical gentamicin 1.5% were prescribed. The patient was reviewed daily to closely monitor changes and to ensure treatment compliance. On successive review, the previously visualised epithelial defect had increased in size significantly, now encompassing almost the entire anterior surface of the cornea. In addition, a localised area of corneal perforation, which appeared to be self-sealed with prolapsed iris, was now visualised on the superior cornea for the first time. The anterior chamber was flat and a 2 mm hypopyon was visible.

On 8th August 2020, the microbiologist confirmed our corneal scraping had identified the rare bacterium Corynebacterium bovis using matrix-assisted laser desorption/ionisation time-of-flight mass spectrometry (MALDI-TOF MS). Initial sensitivities to ciprofloxacin, vancomycin, clindamycin, rifampicin and resistance to penicillin were determined using European Committee on Antimicrobial Sensitivity Testing (EUCAST) clinical breakpoints [13]. The corneal scraping also isolated the organisms *Staphylococcus epidermidis* and *Candida parapsilosis* by way of direct culture. Due to the rarity of *Corynebacterium bovis*, and previous literature reporting pathogenicity predominantly in cattle, we proceeded to enquire about any recent animal contact. Our patient confirmed that she was recently feeding cattle near her residence. On the advice of our microbiology colleagues, we discontinued gentamicin 1.5% eye drops and added vancomycin 5% eye drops hourly with oral ciprofloxacin 750 mg twice daily. On this treatment regimen, the clinical presentation improved significantly, with a reduction in the size of the stromal infiltrate and improved visualisation of the underlying anterior chamber structures noted. Despite this, however, the previously visualised epithelial defect and stromal thinning had again progressed in size.

**Figure 1:**
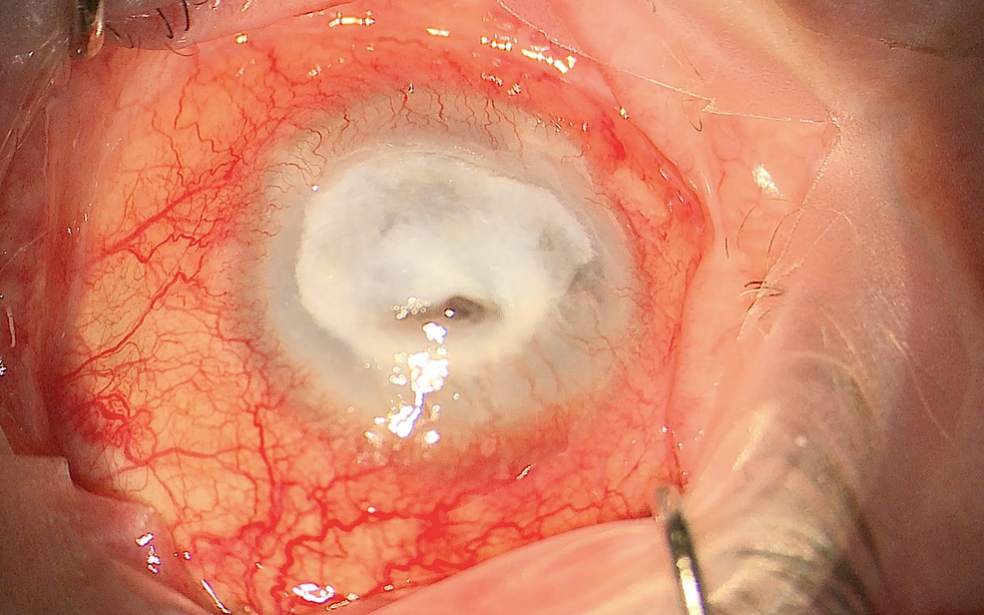
Large epithelial defect with dense stromal infiltrate. A colour photograph showing a large central epithelial defect covered with dense white infiltrate, and a superior localised area of perforation with iris prolapse.

**Figure 2:**
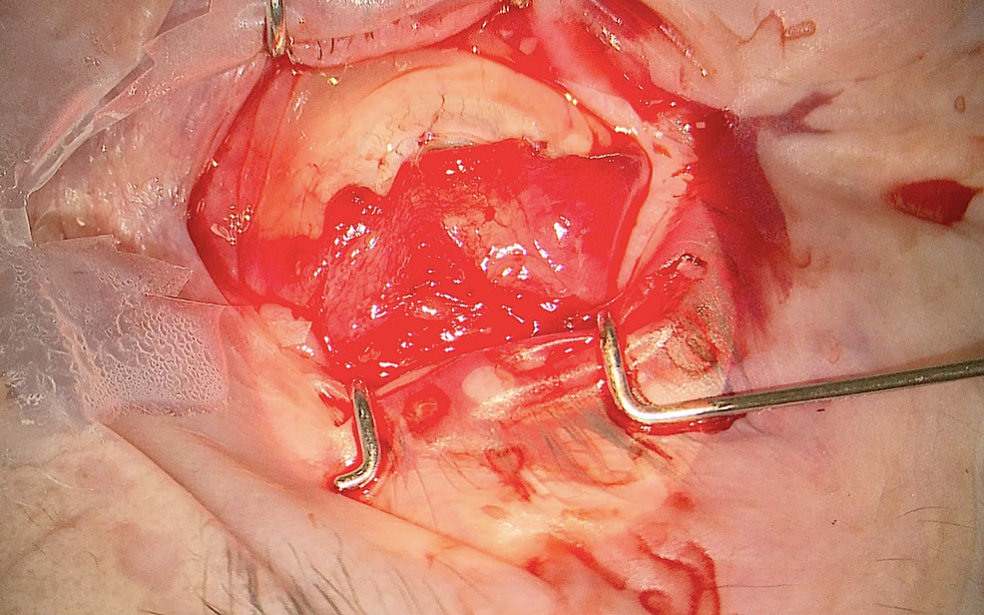
Gundersen conjunctival flap. A colour photograph showing the conjunctival flap secured onto the anterior surface of the cornea.

By 15th September 2020, the BCVA had deteriorated to perception of light. Due to the persisting localised perforation and very large central epithelial defect (Figure 1), we decided to perform Gundersen conjunctival flap surgery with further intraoperative corneal scraping. Iridectomy of the prolapsed iris was performed and cyanoacrylate glue was subsequently utilised to seal the corneal perforation. We then proceeded to enclose the cornea with a conjunctival flap (Figure 2). Postoperatively, the patient reported a substantial reduction in pain. Intraoperative corneal scraping did not isolate any organisms and in view of this, we decided to taper the antibiotic medications, commence topical nepafenac four times daily and continue the lubricating agents. During subsequent one-year follow-up of our patient, BCVA was noted to be perception of light; however, the patient reports no pain in this eye, and remains comfortable with the use of topical lubricating agents as and when required. 

## Discussion

The genus Corynebacterium was first described by Lehman and Neumann in 1877 with their principal features demonstrated by Collins and Cummins in 1986 [5]. They are characterised by non-spore-forming, nonencapsulated curved or straight rods, commonly forming bifurcated aggregations in culture, often referred to as ‘Chinese letters’ [5]. Corynebacterium species have previously been reported as normal commensals of the conjunctival sac. This, however, renders their pathogenicity in causing ocular infections uncertain [5]. Nevertheless, previous reports have established the role of Corynebacterium species in ocular infection, especially in immunocompromised settings [3-6]

*Corynebacterium bovis* is a facultatively anaerobic, catalase-positive, Gram-positive bacterium, which forms part of the Corynebacterium genus. It is a frequent cause of bovine mastitis, however, it is rarely known to cause human infection, and more specifically ocular infection [12]. Despite this, previous literature has reported a role in causing keratitis, but the incidence of keratitis secondary to *Corynebacterium bovis* appears to be significantly less than that of other Corynebacterium species [5].

An interesting feature of this case was the improvement in the clinical picture following the addition of vancomycin 5% eye drops to the treatment regimen. Fluoroquinolone antibiotics offer broad-spectrum activity in treating microbial keratitis, hence they are frequently used as first-line therapy in the treatment of microbial keratitis. Despite the initial sensitivities to ciprofloxacin in this case, antibiotic monotherapy consisting of solely fluoroquinolones may cause inadequate treatment of this bacterium, with consequent complications such as corneal perforation as in this case.

The confirmation of recent cattle contact by our patient suggests that the source of this rare organism was indeed hand-to-eye contact from infected cattle. Although commonly resulting in bovine infiltration and subsequent infection, this case indicates a likely transmission route of *Corynebacterium bovis* from cattle to humans via direct contact or via fomites, with subsequent pathogenicity.

Furthermore, there has yet to be any reports detailing the potential severity of corneal infection secondary to Corynebacterium bovis infiltration. This case report highlights the pathogenicity of *Corynebacterium bovis* in severe cornea infection, particularly in locally immunocompromised environments. 

## Conclusions

To the best of our knowledge, this is the first reported case of profuse bacterial keratitis, on a background of viral keratitis, resulting in corneal perforation with *Corynebacterium bovis* infiltration. This report highlights both the clinical and management challenges endured in treating this rare cause of bacterial keratitis and describes various characteristics of this unusual pathogen.
